# Serum procalcitonin measurement as diagnostic and prognostic marker in febrile adult patients presenting to the emergency department

**DOI:** 10.1186/cc5926

**Published:** 2007-05-23

**Authors:** Pierre Hausfater, Gaëlle Juillien, Beatrice Madonna-Py, Julien Haroche, Maguy Bernard, Bruno Riou

**Affiliations:** 1 Service d'Accueil des Urgences, Centre Hospitalier Universitaire Pitié-Salpêtrière, Assistance Publique-Hôpitaux de Paris, Université Pierre et Marie Curie-Paris 6, 47-83 Boulevard de l'Hôpital, 75651 Paris Cedex 13, France; 2 Service de Médecine Interne, Centre Hospitalier Universitaire Pitié-Salpêtrière, Assistance Publique-Hôpitaux de Paris, Université Pierre et Marie Curie-Paris 6, 47-83 Boulevard de l'Hôpital, 75651 Paris Cedex 13, France; 3 Fédération de Biochimie, Centre Hospitalier Universitaire Pitié-Salpêtrière, Assistance Publique-Hôpitaux de Paris, Université Pierre et Marie Curie-Paris 6, 47-83 Boulevard de l'Hôpital, 75651 Paris Cedex 13, France

## Abstract

**Introduction:**

Identification of bacterial infections is crucial if treatment is to be initiated early and antibiotics used rationally. The primary objective of this study was to test the efficiency of procalcitonin (PCT) in identifying bacterial/parasitic episodes among febrile adult patients presenting to an emergency department. Secondary objectives were to identify clinical or biological variables associated with either bacterial/parasitic infection or critical illness.

**Methods:**

This was a prospective, single centre, non-interventional study, conducted in the adult emergency department of an academic tertiary care hospital. We included patients with body temperature of 38.5°C or greater. A serum sample for measurement of PCT was collected in the emergency room. Patients were followed up until day 30. After reviewing the medical files, two independent experts, who were blind to the PCT results, classified each of the patients as having a bacterial/parasitic infection, viral infection, or another diagnosis.

**Results:**

Among 243 patients included in the study, 167 had bacterial/parasitic infections, 35 had viral infections and 41 had other diagnoses. The PCT assay, with a 0.2 μg/l cutoff value, had a sensitivity of 0.77 and a specificity of 0.59 in diagnosing bacterial/parasitic infection. Of the patients with PCT 5 μg/l or greater, 51% had critical illness (death or intensive care unit admission) as compared with 13% of patients with lower PCT values.

**Conclusion:**

Bearing in mind the limitations of an observational study design, the judgements of the emergency department physicians were reasonably accurate in determining the pretest probability of bacterial/parasitic infection. PCT may provide additional, valuable information on the aetiology and prognosis of infection in the emergency department.

## Introduction

Accurate identification of bacterial aetiology of fever in patients attending the emergency department (ED) is a desirable objective but it is often unattainable, largely because signs and symptoms of bacterial and viral infections overlap considerably. Delay in identifying pathogens from specimen cultures adds to the difficulty in establishing an aetiological diagnosis in the ED and leads to inappropriate use of antibiotics. In addition, estimation of the severity of bacterial infection is mostly based on the presence of characteristics suggestive of systemic inflammatory response syndrome, which may not be apparent when the patient is seen early in the course of the infection.

Procalcitonin (PCT) concentration is raised in the serum of patients with severe bacterial infection [1-4]. The primary objective of our study was to test the efficiency of PCT in identifying bacterial/parasitic episodes among febrile adult patients presenting to an ED. Secondary objectives were to identify clinical or biological variables associated with either bacterial or parasitic infection, and to identify clinical or biological variables associated with critical illness during the febrile episode.

## Materials and methods

### Patients

Patients were eligible for inclusion in this prospective study if they presented, during the period from 1 June 2003 to 29 February 2004, with a oral temperature of 38.5°C or greater to the adult ED of a 1800-bed academic tertiary care hospital. Patients younger than 15 years were excluded. Immunocompromised status was not an exclusion criterion. The study was approved by the ethical committee of our hospital (Comité de Protection des Personnes Pitié-Salpêtrière, Paris, France). All patients gave written informed consent.

After evaluation in the emergency room, each patient was examined by a senior physician. Blood sampling and radiological examinations were ordered in accordance with routine care apart from blood chemistry, which in all patients included a sample for PCT measurement. Microbiological work up varied according to infectious clinical focus but included, in all patients, one pair of blood culture and urinary analysis. The emergency physician completed a standardized form for each patient, including co-morbidity, vital signs, putative source of infection, prior antibiotic therapy before ED consultation, and presence or absence of headache, abdominal pain, diarrhoea, myalgia, sore throat, rhinorrhoea, dry cough, polyadenopathy and rash. The form ended with the emergency physician's diagnostic suspicion, antibiotic prescription and patient course (not admitted or admitted to a medical, surgical, or intensive care unit [ICU] bed). PCT results were not available at this time.

All patients were followed up by study investigators at 8 and 30 days, either via hospital medical files or after a telephone call in those patients not admitted or with short hospital stay. Follow-up assessment included the following factors: fever resolution (either spontaneously or with antibiotics), change in final diagnosis, occurrence of another infectious phase or hospital stay, and outcome (deceased or alive).

Two independent experts, who were blind to the PCT results, reviewed each complete medical history and categorized patients into one of three groups: bacterial or parasitic infection; viral infection; or other. In case of disagreement between the two experts, consensus was reached with a third expert. Bacterial and parasitic infections were pooled together because both are associated with significant elevations in PCT levels as compared with viral infections. The category 'other' included non-infectious aetiologies of fever. The viral category was divided into acute (for instance, influenza) and chronic (for example, HIV, or hepatitis B or C virus) viral infections. The febrile episode was attributed by experts to a viral aetiology for all cases of acute viral infection. Chronic viral infection was retained as the aetiology of febrile episode after all other causes of fever had been ruled out.

This classification by the experts was considered the 'gold standard' for fever aetiology, against which PCT measurement was evaluated.

### Procalcitonin assay

For serum PCT measurement, we used a time-resolved amplified cryptate emission technology assay (Kryptor PCT, Brahms, Hennigsdorf, Germany). This assay is based on a polyclonal antibody against calcitonin and a monoclonal antibody against katacalcin, which bind to the calcitonin and katacalcin sequence of precursor molecules. This assay has an optimized functional sensitivity of 0.06 μg/l. In healthy volunteers, normal PCT levels are under 0.1 μg/l. Based on previous studies involving ED patients, we chose a cutoff of 0.2 μg/L for the PCT assay [2,5].

### Statistical analysis

Data are expressed as mean ± standard deviation or median and 95% confidence interval (CI) for non-Gaussian distributions. Comparison of two means was performed using the Student's *t*-test, comparison of two medians using the Mann-Whitney test, and comparison of two proportions using the Fisher's exact method.

Sensitivity, specificity, positive and negative predictive values, and accuracy (defined as the sum of concordant cells divided by the sum of all cells in a 2 × 2 table) and their 95% CIs were calculated. Comparisons of these diagnostic variables between PCT and emergency physician were performed using the CI method.

We assessed the associations between various variables, including the diagnosis suspected by the emergency physician, elevated PCT, and the final diagnosis of the experts (bacterial/parasitic and viral infection) using a stepwise forward logistic regression. We also entered all variables with a univariate *P *value below 0.05 into the model. We considered variables with a multivariate *P *value below 0.05 to be independent indicators of bacterial/parasitic infection and retained them in the model. Continuous variables were transformed into dichotomous variables using the receiver operating curve (ROC); specifically, we determined the optimal threshold that minimized the distance to the ideal point (sensitivity = specificity = 1) on the ROC curve. The discrimination ability of the final model was quantified by using the area under the ROC curve with its 95% CI. The calibration of the final model was assessed using the Hosmer-Lemeshow goodness-of-fit test. The same procedure was applied to determine the variables associated with critical illness, defined as admission into an ICU or death within 30 days.

All statistical comparisons were two-tailed, and a *P *value of less than 0.05 was required to reject the null hypothesis. Statistical analysis was performed using a computer and NCSS 2001 software (Statistical Solutions Ltd, Corke, Ireland).

## Results

**Figure 1:**
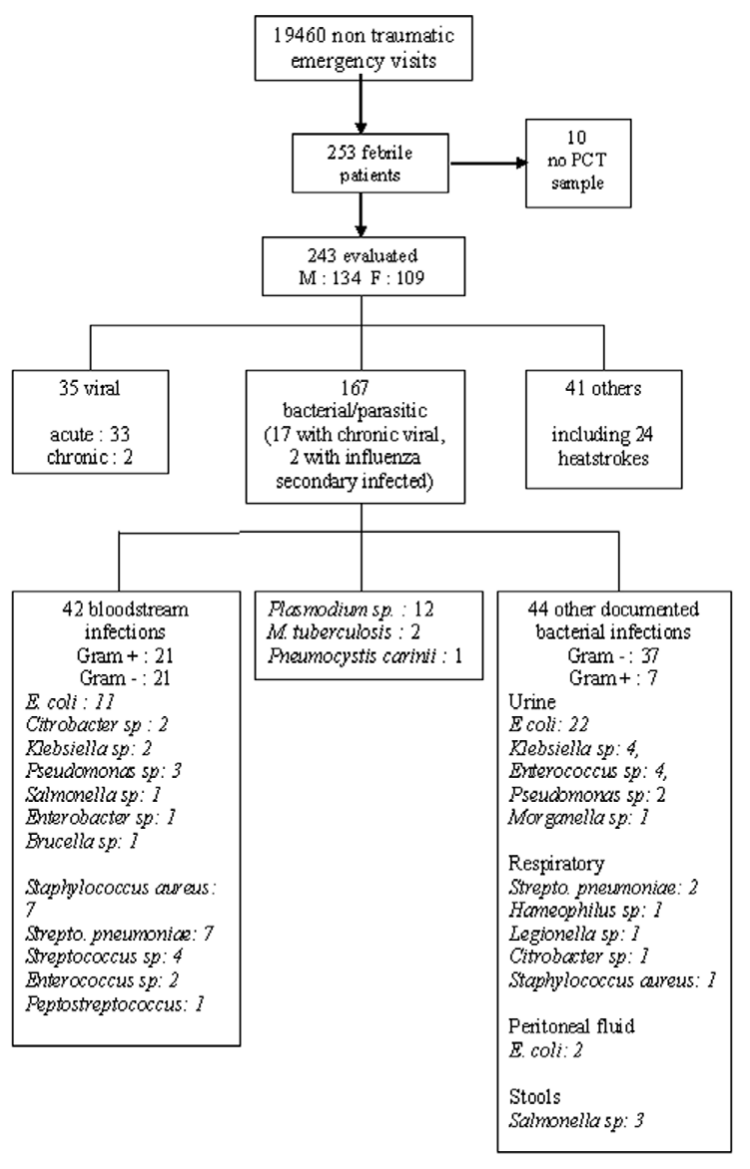
Study design flow diagram and outcomes. F, female; M, male; PCT, procalcitonin.

The flow diagram of the study is depicted in Figure 1. Among the 19,460 atraumatic patients who were examined in our ED during the study period, we included 253 (1.3%) consecutive febrile patients. Ten patients were excluded from the analysis because PCT samples were lacking. Baselines characteristics of the remaining 243 patients were as follows: 134 were male and 109 were female, and the mean age was 56 ± 21 years. Seventy-one patients (29%) were immunocompromised: 22 patients were being treated for solid organ tumours, 19 had chronic HIV infection, 17 were being treated for malignant haemopathy, five had undergone solid organ transplantation, three were on immunosuppressive therapy, two were on corticosteroid therapy, two had undergone splenectomy and one had Down's syndrome. Thirty-two patients (13%) were receiving antibiotics when they presented at the ED. Overall, 196 (81%) patients were admitted to hospital (median duration 7 days, 95% CI 6 to 8 days), of which 31 (13%) were admitted to the ICU (immediately after ED evaluation in 24 patients and during the first 48 hours in seven). Blood culture was performed in 237 (98%) patients, and of these 42 (18%) cultures exhibited growth of pathogenic micro-organisms (Gram positive, 21; Gram negative, 21; both Gram positive and negative, 2). The majority of patients (83%) left the ED with an antibiotic prescription. Twenty-three patients (10%) were deceased on day 8, and 30 (13%) patients were deceased on day 30.

According to the expert classification, 167 (69%) patients had bacterial/parasitic infection, including 17 with concomitant chronic viral infection, and two with secondary influenza infection. Thirty-five patients (14.5%) had a viral infection; in 33 of these patients the infection was acute and in two it was chronic (without an alternative aetiology for the fever). A total of 41 patients were deemed to be in the 'other' category, including 24 patients with environmental heatstroke (four with chronic viral infection), two with acute pancreatitis, and one patient with haemophagocytic syndrome. Parasitic infections were malaria (in 12 patients) and *Pneumocystis carinii *pneumonia (in one patient). The main diagnoses of the patients are summarized in Table 1. Agreement between the two experts occurred in 89% of cases for the diagnosis of bacterial/parasitic infection and in 93% of cases for the diagnosis of viral infection.

**Table 1:** Main clinical diagnoses of the 243 patients established after expert evaluation (blinded to PCT results)

Major diagnosis	Number of patients (%)	Aetiology (*n *[%])		
		
		Bacterial	Viral	Parasitic
Infection	202 (83)	154 (76)	35 (17)	13 (7)
Respiratory tract infection	69 (28)	62 (90)	6 (8.5)	1 (1.5)
Urinary tract infection	42 (21)	42	-	-
Digestive tract infection	19 (9.4)	15 (79)	4 (21)	-
Flu-like	14 (7)	-	14	-
Malaria	12 (6)	-	-	12
Bacteraemia of unknown origin	12 (6)	12	-	-
Ear nose and throat infection	9 (4.5)	5 (55)	4 (45)	-
Wound and soft tissue infection	7 (3.5)	7	0	-
Bone and joint infection	3 (1.5)	3		
Peritonitis	3 (1.5)	3	0	-
Viral meningitis	4 (1.3)	-	4	-
Purulent meningitis	2 (2)	2	-	-
Endocarditis	2 (2)	2	-	-
Brucellosis	1 (0.5)	1	-	-
Chronic viral infection	2 (2)	-	2	-
Herpes simplex	1 (0.5)	-	1	-

Non-infectious febrile episodes	41 (17)			
Febrile neutropenia and	7	-	-	-
Neoplasic fever				
Heatstroke	24	-	-	-
FUO	3	-	-	-
Acute pancreatitis	2	-	-	-
Haemophagocytic syndrome	1	-	-	-
Alcoholic hepatitis	1	-	-	-
Systemic vasculitis	1	-	-	-
Drug hypersensitivity syndrome	1	-	-	-
Acute appendicitis	1	-	-	-

Total	243 (100)			

FUO, fever of unknown origin; PCT, procalcitonin.

### Diagnosis of bacterial/parasitic infection

**Figure 2:**
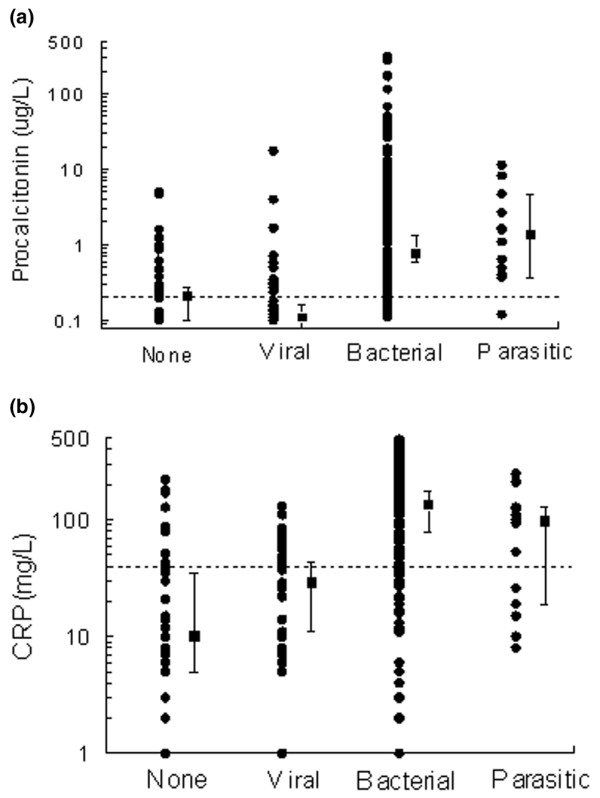
Values of biomarkers in the different febrile groups: none (noninfectious), and viral, bacterial and parasitic infections. **(a) **Procalcitonin (PCT). **(b) **C-reactive protein (CRP). Data are presented in a semi-logarithm scale. Each dark circle indicates a patient. Dark squares represent medians (95% confidence interval) and dotted lines the optimal threshold determined on receiver operating characteristic curve: 0.2 μg/l for PCT and 40 mg/l for CRP.

PCT concentrations were above 0.2 μg/l in 128 out of 173 (74%) patients with bacterial/parasitic infections. The diagnostic performance of PCT is shown and compared with that of the emergency physician in Table 2. For PCT, the area under ROC curve was 0.766 (95% CI 0.697 to 0.821; *P *< 0.001) for the diagnosis of bacterial/parasitic infection. Individual values for PCT and C reactive protein are represented in Figure 2. Based on the ROC curve, the optimal threshold for PCT was confirmed to be 0.2 μg/l. When the subgroup of patients with positive blood cultures or positive *Plasmodium *thick smear tests was considered, PCT had a sensitivity of 0.87 (95% CI 0.76 to 0.94), a specificity of 0.41 (95% CI 0.34 to 0.48), a positive predictive value of 0.30 (95% CI 0.24 to 0.38) and a negative predictive value of 0.92 (95% CI 0.83 to 0.96). The accuracy of PCT was found to be 0.50 (95% CI 0.44 to 0.56) in predicting bloodstream infection.

**Table 2:** PCT and CRP versus emergency physician judgement in diagnosing bacterial/parasitic infection

Test and cutoff	Sensitivity (95% CI)	Specificity (95% CI)	PPV (95% CI)	NPV (95% CI)	Accuracy (95% CI)
CRP					
≥ 5 mg/l	0.96 (0.91–0.98)	0.16 (0.09–0.26)	0.71 (0.65–0.77)	0.63 (0.41–0.81)	0.71 (0.64–0.76)
≥40 mg/l	0.76 (0.69–0.82)*	0.62 (0.51–0.72)	0.81 (0.74–0.87)	0.54 (0.44–0.64)*	0.71 (0.65–0.77)
≥100 mg/l	0.54 (0.46–0.62)	0.90 (0.82–0.95)	0.93 (0.85–0.96)	0.47 (0.39–0.56)	0.65 (0.59–0.71)
PCT					
≥0.1 μg/l	0.90 (0.85–0.94)	0.32 (0.22–0.43)	0.74 (0.68–0.80)	0.60 (0.47–0.74)	0.72 (0.66–0.77)
≥0.2 μg/l	0.77 (0.70–0.82)*	0.59 (0.48–0.70)	0.80 (0.74–0.86)	0.54 (0.43–0.64)*	0.71 (0.65–0.77)
≥0.5 μg/l	0.63 (0.55–0.70)	0.79 (0.68–0.87)	0.87 (0.80–0.92)	0.49 (0.40–0.58)	0.68 (0.62–0.73)
≥2 μg/l	0.36 (0.30–0.44)	0.93 (0.85–0.97)	0.92 (0.83–0.97)	0.40 (0.33–0.47)	0.54 (0.48–0.60)
≥5 μg/l	0.23 (0.17–0.30)	0.99 (0.93–1.00)	0.97 (0.87–0.99)	0.37 (0.30–0.44)	0.46 (0.40–0.53)
Emergency physician	0.85 (0.79–0.90)	0.57 (0.45–0.67)	0.81 (0.75–0.86)	0.63 (0.51–0.74)	0.76 (0.70–0.81)

Shown is a comparison of performance of procalcitonin (PCT) and C-reactive protein (CRP) with emergency physician for the diagnosis of bacterial/parasitic infection, with the 'gold standard' being experts diagnosis. *P < 0.05, versus emergency physician. CI, confidence interval; NPV, negative predictive value; PPV, positive predictive value.

Patients with bacterial infection who were receiving antibiotics when attending the ED were compared with patients who were not. Total white blood cell count, neutrophil leucocytes and C-reactive protein values did not differ significantly between groups. Median PCT concentrations were similar in both groups, at 0.78 μg/l (95% CI 0.49 to 3.80 μg/l) and 0.79 μg/l (95% CI 0.56 to 1.47 μg/l), respectively (not significant). The percentage of patients with elevated PCT did not differ significantly between groups (77% versus 76%).

Finally, immunocompromised patients were compared with the immunocompetent group. First, there was no difference between the number of bacterial/parasitic episodes (according to expert classification) in the two groups, at 51 (72%) versus 116 (67%), respectively (*P *= 0.55, not significant). The PCT area under ROC curve was not statistically different between immunocompromised and immunocompetent groups, at 0.792 (95% CI 0.654 to 0.879) and 0.759 (95% CI 0.673 to 0.854), respectively (*P *= 0.63, not significant). However, PCT values were significantly higher in immunocompromised than in immunocompetent patients, at 1.85 μg/l (95% CI 0.67 to 4.70 μg/l) versus 0.61 μg/l (95% CI 0.38 to 1.10 μg/l; *P *= 0.005), respectively, for patients with bacterial/parasitic infection, and 0.26 μg/l (95% CI 0.15 to 0.5 μg/l) versus 0.11 (95% CI 0.05 to 0.14; *P *= 0.01) for patients without bacterial/parasitic infection.

Stepwise logistic regression analysis showed four independent variables to be significantly associated with a diagnosis of bacterial/parasitic infection (Table 3): emergency physician suspicion, C-reactive protein over 40 mg/l, neutrophil leucocytes over 7,500/mm^3^, and PCT over 0.2 μg/l (Table 3).

**Table 3:** Comparison of patients with or without bacterial/parasitic infection (univariate analysis) and identification of variables predictive of bacterial/parasitic infection after stepwise logistic regression analysis (multivariate analysis)

Variable	Univariate analysis			Multivariate analysis	
	
	Nonbacterial/parasitic (*n *= 76)	Bacterial/parasitic (*n *= 167)	*P*	Odds ratio [95% CI]	*P*
Sex (male)	40 (47%)	94 (44%)	NS		
Age (year)	54 ± 25	57 ± 19	NS		
Temperature (°C)	39.1 ± 0.6	39.2 ± 0.6	NS		
Heart rate (beats/min)	101 ± 21	107 ± 20	0.03		
Systolic arterial blood pressure (mmHg)	131 ± 23	127 ± 22	NS		
Immunodepression	20 (26%)	51 (30%)	NS		
Headache	27 (35%)	37 (22%)	0.04		
Myalgia	23 (30%)	30 (18%)	0.04		
Emergency physician diagnosis	70 (29%)	173 (71%)	< 0.001	7.54 [3.60–15.82]	< 0.001
Haemoglobin level (mg/l)	128 ± 19	125 ± 23	NS		
White blood cell count (/mm^3^)	8060 ± 3777	11688 ± 8039	< 0.001		
Neutrophil leukocytes ≥ 7,500/mm^3^	21 (28%)	88 (54%)	< 0.001	3.17 [1.52–6.62]	0.002
Platelet count (10^3^/mm^3^)	198 ± 90	204 ± 99	NS		
Creatinine (μmol/l)	97 ± 39	118 ± 97	NS		
PCT (μg/l)	0.7 ± 2.2	11.1 ± 39.0	< 0.001		
PCT ≥ 0.2 μg/l	31 (41%)	128 (77%)	< 0.001	4.54 [2.19–9.39]	< 0.001
CRP (mg/l)	39 ± 48	150 ± 128	< 0.001		
CRP ≥ 40 mg/l	28 (38%)	122 (76%)	< 0.001	3.67 [1.79–7.53]	< 0.001

Data are expressed as mean ± standard deviation or number (%). For multivariate analysis all other *P *values were NS. CI, confidence interval; CRP, C-reactive protein; NS, not significant; PCT, procalcitonin.

Among 173 patients with bacterial/parasitic infection (according to emergency physician diagnosis), antibiotic treatment was not initiated in emergency room in nine (5%) patients, although five of them had positive PCT values, including three with positive blood cultures. Conversely, among the 20 patients with acute viral infection according to the emergency physician diagnosis, seven (35%) were given antibiotics.

### Prediction of critical illness

**Figure 3:**
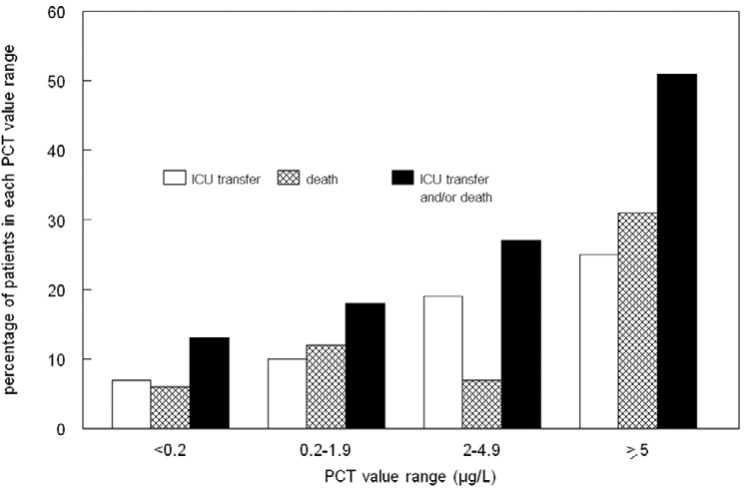
Relation between critical illness (death or ICU transfer) and PCT value range. A total of 55 patients had critical illness, 31 of which were intensive care unit (ICU) transfers and 30 died (including six patients admitted to the ICU). PCT, procalcitonin.

At 30 days of follow up, 55 patients presented with critical illness (31 admissions to the ICU and 30 deaths, including six patients admitted to the ICU). Two of the six patients with bacterial infections, initially admitted to a medical bed and transferred within the 48 subsequent hours to the ICU, had a PCT level above 5 μg/l (8.53 μg/l and 282 μg/l). Similarly, three patients with bacterial infection were initially admitted to a medical bed and died suddenly before day two. None of these patients had co-morbidity that could have restricted ICU transfer (for instance, end-stage cancer or severe neurodegenerative disease) and all three had PCT concentrations above 5 μg/l (5.8, 36 and 316 μg/l). Univariate analysis showed that age, body temperature, white blood cell count, C-reactive protein and PCT were significantly greater in patients with critical illness, as were associated immunocompromised status and altered haemodynamic or renal function at ED assessment. Stepwise logistic regression analysis showed that three variables were significantly associated with critical illness: PCT of 2 μg/l or greater, heart rate above 120 beats/min, and creatininaemia of 120 μmol/l or greater (Table 4). Moreover, there was a correlation between the magnitude of elevation in PCT and the likelihood of subsequent critical illness or death (Figure 3). Indeed, 51% of patients with PCT concentrations of 5 μg/l or greater were deceased at day 30 or admitted to the ICU, as compared with 13% of patients in whom PCT concentration was not elevated.

**Table 4:** Univariate analysis of patients deceased at day 30 or admitted to ICU versus all others patients, and identification of variables associated with critical illness after stepwise logistic regression analysis

	Univariate analysis			Multivariate analysis	
	
	Group II (*n *= 166)	Group I (*n *= 55)	*P*	Odds ratio [95% CI]	*P*
Male sex	91 (54%)	33 (60%)	NS		
Age (year)	55 ± 22	65 ± 16	0.001	1.02 [1.00–1.04]	0.013
Temperature (°C)	39.1 ± 0.6	39.4 ± 0.7	0.01		
Heart rate > 120 beats/min	33 (20%)	19 (34%)	0.03	2.36 [1.22–4.96]	0.02
Systolic blood pressure < 90 mmHg	1 (1%)	3 (5%)	0.047		
White blood cell count (/mm^3^)	10272 ± 7195	26601 ± 83080	0.01		
Previous antibiotic treatment	24 (14%)	7 (13%)	NS		
Antibiotics initiated in ED	129 (79%)	47 (85%)	NS		
Immunocompromised status	44 (26%)	24 (44%)	0.02		
Positive blood culture or thick smear (*Plasmodium*)	33 (20%)	18 (33%)	NS		
Platelet count (10^3^/mm^3^)	199 ± 93	194 ± 102	NS		
Haemoglobin level (mg/l)	12.8 ± 2	12 ± 2.8	0.02		
CRP (mg/l)	103 ± 112	149 ± 144	0.01		
Creatinine ≥ 120 μmol/l	29 (17%)	23 (42%)	< 0.001	2.16 [1.04–4.49]	0.04
PCT μg/l	3.1 ± 10.8, 0.3 [0.3–0.5], (0–115)	24.3 ± 63.7, 1.9 [0.8–4.7], (0–316)	< 0.001		
PCT ≥ 2 μg/L	36 (22%)	27 (49%)	< 0.001	2.51 [1.25–5.04]	0.001

Shown is a univariate analysis comparing group I (patients deceased at day 30 or admitted to intensive care unit [ICU]) and group II (all other patients) and identification of variables associated with critical illness (ICU admission or death) after stepwise logistic regression analysis. Data are expressed as number of patients (%), mean ± standard deviation, median [95% CI] (extrems). For multivariate analysis all other *P *values were NS. CI, confidence interval; CRP, C-reactive protein; ED, emergency department; NS, not significant; PCT, procalcitonin.

## Discussion

The main purpose of the present study was to test the efficiency of PCT in identifying bacterial/parasitic episodes among febrile adult patients presenting to an ED. Therefore, a limitation of the present study is that we did not include febrile outpatients who were normothermic in the ED because of ongoing antipyretic drugs treatment. Second, we did not study PCT kinetics in infected patients with low initial PCT levels. In practice, however, ED physicians must base their biologic evaluation on a single blood sample and not on sequential samples. Unlike the majority of studies published to date on the PCT assay, we did not focus on an organ-specific infection, as well we did not exclude immunocompromised patients [5-10]. Fever does not represent a predominant symptom among patients attending an ED, accounting for only 1.7% to 2.5% of them [11,12]. During the duration of the present study, the patients included accounted for 1.3% of the total number of medical emergency consultations, assuming that the majority of febrile patients had actually been screened. The population that we studied probably reflects the broad aetiological diagnoses and situations that may confront emergency physicians when they attend to adult febrile patients.

The major limitation of our study is the choice of gold standard against which to assess the aetiology of febrile episodes. We considered expert diagnosis to be more suitable, because a significant proportion of bacterial febrile episodes is never confirmed microbiologically in the setting of ED care. Although such methodology might have led to misclassification, agreement between the two experts was good (89% to 93%).

PCT at a cutoff value of 0.2 μg/l exhibited a sensitivity of 0.77 and a specificity of 0.59 for identifying patients with bacterial/parasitic infection, and its efficiency was comparable to that of the emergency physician (Table 2). Moreover, among the biologic variables evaluated, PCT was found to be the most predictive of bacterial/parasitic infection, with an odds ratio of 4.54 (Table 3). In a previous study, the same PCT cutoff value was associated with a 0.62 sensitivity and 0.88 specificity [2]. However, the population studied was not the same as that in the present study, in which only feverish patients were included; therefore, the prevalence of bacterial infection in that previous study was much lower. Moreover, in that previous study [2] we used a less sensitive assay for PCT measurement (LUMitest PCT [Brahms Diagnostica]; functional sensitivity 0.33 μg/l), which could have underestimated the number of patients with low concentrations. Similarly, using the same less sensitive assay (LUMItest) and a PCT cutoff value of 0.6 μg/L, Chan and coworkers [13] reported a 0.69 sensitivity and a 0.65 specificity for PCT in identifying infection among unselected atraumatic patients admitted via their ED. In a pediatric population, Gendrel and colleagues [4] reported on the ability of a PCT assay to identify bacterial infection, and found a 0.83 sensitivity and a 0.93 specificity with a cutoff PCR value of 1 μg/l. However, that study was restricted to febrile children in whom a responsible pathogen was identified, leading to exclusion of more than 50% of patients initially screened. In another pediatric study conducted in children presenting with body temperature above 38°C [14], sensitivity and specificity of PCT were 0.65 and 0.94, respectively, for a 0.53 μg/l cutoff.

The lower specificity that we report (0.59) may have two primary explanations. First, we retained a low cutoff value for PCT assay (0.2 μg/l). This cutoff value was retained based on statistical considerations because it was the optimal compromise between sensitivity and specificity on the ROC curve. However, in practice the threshold must be suited to the clinical context. For example, on one hand, a 0.1 μg/l cutoff may be useful in immunocompromised patients to screen for bacterial infection, although it may lead to a significant proportion of false-positive results (specificity 0.32; Table 2). On the other hand, a 2 μg/l threshold exhibited excellent specificity for bacterial infection (0.93), but it lacked sensitivity (0.36), which would lead to under-diagnosis a large proportion of infected patients (Table 2). Because a 0.25 μg/l threshold was reported to be useful within the context of respiratory tract infection [5,15] and in the ED setting [2], our choice of a 0.2 μg/l cutoff in the present study is a rational one. Finally, because PCT determination in ED is performed early in the course of infection, a low threshold may be more appropriate in this setting than the higher threshold that could be used in the ICU. However, further studies are required to determine precisely the optimal PCT thresholds for application in febrile patients presenting to the ED. The second reason that may account for the the lower specificity we report here is that of the 31 patients with no bacterial/parasitic infection but with raised PCT concentrations (false positives), 12 had diseases that have previously been shown to be associated with elevated PCT, namely acute pancreatitis, environmental heatstroke and haemophagocytic syndrome [4,16,17].

The PCT assay was particularly efficient at identifying critical bacterial/parasitic infections. First, the sensitivity of PCT was higher (0.87) for the diagnosis of bacteraemic infection, and a PCT value under 0.2 μg/l made bloodstream infection unlikely (negative predictive value 0.92). These findings are in accordance with a previous study [18] reporting a negative predictive value of 0.99 for a PCT cutoff value of 0.4 μg/l, when comparing bacteraemic with nonbacteraemic infectious episodes among patients hospitalized for community-acquired infection. Using the same 0.2 μg/l cutoff value as in the present study, Caterino and coworkers [19] identified a 0.93 sensitivity and a 0.38 specificity for PCT assay in detecting bacteraemia in older ED patients. Second, the magnitude of the rise in PCT correlated with the severity of infectious disease (Figure 3). Indeed, more than half of the patients with PCT above 5 μg/l in the emergency room were either transferred to the ICU or had died by day 30. For five patients initially admitted to a medical ward but who subsequently died or were admitted to the ICU, the availability of PCT results in the emergency room might have helped the emergency physician to identify the severity of illness and to opt for ICU admission. The efficiency of PCT in predicting critical illness has already been reported in pediatric, adult ICU and, less frequently, ED populations, with cutoff values between 3 and 33 μg/L [1-3,20-23]. However, because most previous studies were conducted in an ICU setting, our study does support the usefulness of PCT in the emergency room in identifying those patients in whom early ICU admission may be justified. The place of PCT together with severity scores such as the Pneumonia Severity Index must be defined. Early assessment based on PCT findings might be of paramount importance, because early goal-directed therapy has been proven to confer significant benefit with respect to outcome in patients with severe sepsis and septic shock [24].

Overall, emergency physician judgement appeared to be as efficient as or better than the PCT assay (Table 2). However, usual clinical practice was rather conflicting, in that 193 (83%) patients left the emergency room with antibiotics, although only 173 (71%) were considered to have bacterial/parasitic infection from the emergency physician's point of view. Similarly, seven out of 20 (35%) patients who were considered by the emergency physician to have acute viral infection left the emergency room with antibiotics. Interestingly, the results of two of the three quantitative variables significantly associated with bacterial/parasitic infection were known by emergency physicians and could theoretically have influenced their judgement of etiology. Conversely, one might speculate that knowledge of PCT measurement in some cases of intermediate pre-test probability may turn into high post-test probability of bacterial infection and therefore more suitable care. Biological markers must be considered diagnostic and prognostic tools that should assist physicians in their clinical practice, but they should not replace medical judgement. Within the context of infectious diseases, two recent studies [5,15] pointed out the effectiveness of the PCT assay in safely reducing the number of unnecessary antibiotic prescriptions for management of respiratory tract infections. Although our data support the need for efficient bacterial and viral markers to improve rational use of antibiotics, interventional studies, in which antimicrobial therapy is guided by a marker and in which the primary measure of efficacy is outcome, should be encouraged.

## Conclusion

Within the context of febrile adult patients presenting to an ED, PCT assay at a 0.2 μg/l threshold can help physicians to identify bacterial/parasitic infections. Whether this can back up therapeutic decisions must be investigated in interventional studies. PCT measurement in the emergency room could contribute to the early identification of critical illness. Emergency febrile patients with PCT above 5 μg/l should be carefully monitored to identify severe sepsis or septic shock criteria.

## Key messages

• The optimal PCT threshold in adult febrile patients in the ED may be 0.2 μg/l.

• PCT is an independent variable that can predict whether a febrile episode has a bacterial origin.

• PCT, at a threshold of 2 μg/l, is independently associated with critical illness.

## Abbreviations

CI = confidence interval; ED = emergency department; ICU = intensive care unit; PCT = procalcitonin; ROC = receiver operating characteristic.

## Competing interests

PH received a total of 1500€ for lecture fees in 2006 from BRAHMS France (the manufacturer of the PCT assay). No other author has any competing interest to declare.

## Authors' contributions

PH designed the study, included patients, participated in patient follow up, was an expert in patient classification, conducted data analysis and wrote the paper. GJ included patients, was an expert in patient classification, and conducted the majority of patient follow up. BM-P included patients and participated in patient follow up. JH was an expert in patient classification. MB performed PCT measurements and interpreted the results. BR participated in study design construction, conducted statistical analysis, and participated in data analysis and manuscript writing.
